# Association between thoracic and third lumbar CT-derived muscle mass and density in Caucasian patients without chronic disease: a proof-of-concept study

**DOI:** 10.1186/s41747-023-00340-1

**Published:** 2023-05-29

**Authors:** Mia Solholt Godthaab Brath, Marina Sahakyan, Esben Bolvig Mark, Jens Brøndum Frøkjær, Henrik Højgaard Rasmussen, Lasse Riis Østergaard, Ulla Møller Weinreich

**Affiliations:** 1 Research Unit of Respiratory Diseases, Aalborg University Hospital, Aalborg, Denmark; 2 Department of Clinical Medicine, Aalborg University, Aalborg, Denmark; 3 Department of Respiratory Diseases, Aalborg University Hospital, Aalborg, 9000 Denmark; 4 Department of Radiology, Aalborg University Hospital, Aalborg, Denmark; 5 Department of Gastroenterology and Hepatology, Mech-Sense, Aalborg University Hospital, Aalborg, Denmark; 6 Department of Gastroenterology & Hepatology, Danish Nutrition Science Center, Aalborg University Hospital, Aalborg, Denmark; 7 Department of Gastroenterology & Hepatology, Center of Nutritional and Intestinal Failure, Aalborg University Hospital, Aalborg, Denmark; 8 Medical Informatics Group, Department of Health Science and Technology, Aalborg University, Aalborg, Denmark

**Keywords:** Body composition, Contrast media, Muscle (skeletal), Tomography (x-ray computed)

## Abstract

**Background:**

Computed tomography (CT) is increasingly used in the clinical workup, and existing scan contains unused body composition data, potentially useful in a clinical setting. However, there is no healthy reference for contrast-enhanced thoracic CT-derived muscle measures. Therefore, we aimed at investigating whether there is a correlation between each of the thoracic and third lumbar vertebra level (L3) skeletal muscle area (SMA), skeletal muscle index (SMI), and skeletal muscle density (SMD) at contrast-enhanced CT in patients without chronic disease.

**Methods:**

A proof-of-concept retrospective observational study was based on Caucasian patients without chronic disease, who received CT for trauma between 2012 and 2014. Muscle measures were assessed using a semiautomated threshold-based software by two raters independently. Pearson’s correlation between each thoracic level and third lumbar and intraclass correlation between two raters and test–retest with SMA as proxy parameters were used.

**Results:**

Twenty-one patients (11 males, 10 females; median age 29 years) were included. The second thoracic vertebra (T2) had the highest median of cumulated SMA (males 314.7 cm^2^, females 118.5 cm^2^) and SMI (97.8 cm^2^/m^2^ and 70.4 cm^2^/m^2^, respectively). The strongest SMA correlation was observed between T5 and L3 (*r* = 0.970), the SMI between T11 and L3 (*r* = 0.938), and the SMD between the T10 and L3 (*r* = 0.890).

**Conclusions:**

This study suggests that any of the thoracic levels can be valid to assess skeletal muscle mass. However, the T5 may be most favourable for measuring SMA, the T11 for SMI, and T10 for SMD when using contrast-enhanced thoracic CT.

**Relevance statement:**

In COPD patients, a CT-derived thoracic muscle mass assessment may help identify who would benefit from focused pulmonary rehabilitation: thoracic contrast-enhanced CT conducted as part of the standard clinical workup can be used for this evaluation.

**Key points:**

• Any thoracic level can be used to assess thoracic muscle mass.

• Thoracic level 5 is strongly associated with the 3rd lumbar muscle area.

• A strong correlation between the thoracic level 11 and the 3rd lumbar muscle index.

• Thoracic level 10 is strongly associated with the 3rd lumbar muscle density.

**Graphical Abstract:**

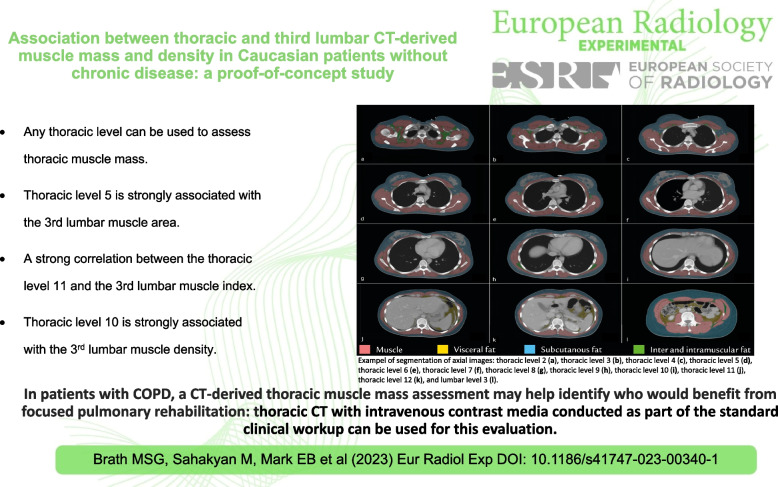

**Supplementary Information:**

The online version contains supplementary material available at 10.1186/s41747-023-00340-1.

## Background

Over the past decades, the importance of body composition has become evident as reduced muscle mass and sarcopenia have adverse outcomes for the patients [1–4]. Reduced muscle mass, quality, and/or strength has been reported to be associated with an increased risk of adverse outcomes such as falls and fractures, more prolonged hospital admissions, increased risk of complications in both medical and surgical treatments, and increased risk of mortality [3–6]. A wide range of different patient groups are at risk of developing reduced muscle mass and/or sarcopenia; these include, among others, diabetes, chronic obstructive pulmonary disease (COPD), and cancer [5–7]. Various metrics are available to determine the body composition, *i.e.*, anthropometric measures such as body mass index (BMI) or body circumferences. However, these do not discriminate between the relative proportions of muscle and fat mass [5], so they do not detect early signs of pathological changes in body composition.

Computed tomography (CT) can differentiate between tissues such as muscle, fat, and bone by their radiodensity. This allows for individual muscles assessment and determine muscle quantity and quality [1, 5, 8]. Conventional abdominal CT scans are widely used in research of body composition, defining typically the third lumbar vertebra (L3) as a reference level of assessment [9, 10]. Based on a single axial image from abdominal imaging, the skeletal muscle mass has been validated and correlated to the overall skeletal muscle mass [11]. The skeletal muscle mass, based on CT, is often reported as the skeletal muscle area (SMA, cm^2^), height-adjusted skeletal muscle index (SMI, cm^2^/m^2^), and skeletal muscle density (SMD, mean Hounsfield unit (HU) of SMA) as a measure of muscle quality [9, 10]. CT scans have become more frequently used and implemented as part of the clinical workup [12, 13]. It has been recommended to use non-contrast CT scans when determining the body composition as there are several considerations when intravenous contrast media is used, *e.g.*, iodine concentration, CT parameters, the circulation of the body, and size of the patient [3, 14]. However, in some patient groups, only thoracic CT is used as part of the standard clinical workup, *e.g.*, in patients with COPD. As patients with COPD are one of the largest, most comorbid patient groups [15–17], there has been an increased focus on the thoracic CT as a source for assessing the skeletal muscle mass as well [18, 19].

In 2021, 296,390 thoracic CT scans were conducted nationwide in Denmark [20]. However, in the daily clinical workup, it is locally seen in the Northern Region of Denmark that 53% of the thoracic CT scans are conducted using intravenous administration of iodine contrast agent (Supplementary Table S1). This is a challenge as there is no reference for contrast-enhanced thoracic CT muscle measure, neither clear reference for conventional abdominal CT, especially on muscle density [21–23]. Before CT scans from the daily clinical work can be used to assess skeletal muscle in patients with chronic diseases, there is a need of a reference of patients without chronic disease with knowledge of sex, age, size, ethnicity, and how contrast-enhanced thoracic CT muscle measures correlate to the L3 muscle measures with the same CT parameters and CT protocol. As such, we want to explore the correlation between muscle quantity and quality indexes on L3 and thoracic levels in patients without chronic disease before measuring them on patients with chronic conditions.

We hypothesised that the thoracic vertebras (T) 2 to T12 in contrast-enhanced thoracic CT were equally suited to assess the muscle mass and density in a Caucasian population without chronic disease. To do so, a systematic image selection and segmentation were conducted from the T2 to T12 with focus on the muscle mass and density.

The primary aim was to investigate whether there is an association between each of the thoracic levels to the L3 level SMA, SMI, and SMD when using contrast-enhanced thoracic and abdominal CT in patients without chronic disease as a proof of concept. The secondary aim was to assess the inter-rater agreement between image selections and inter- and intra-rater agreement on the thoracic skeletal SMA as a proxy parameter.

## Methods

### Study design and population

This retrospective proof-of-concept study was approved by the National Danish Scientific Ethics Committee (2300176), the Danish Patient Safety Authority (31–1521-138), and the Danish Data Protection Agency 2020–154.

The study was carried out in Caucasian patients, who underwent consecutive thoracic and abdominal CT scans as a part of the trauma protocol in the North region of Denmark. Scans were conducted between 2012 and 2014. This period was selected as the trauma protocol was harmonised throughout the region from 2012 and limited to 2014 due to the risk of an unreasonable workload and strict data policies. Patients’ medical records and medical treatment at the time of the scans were assessed.

**Fig. 1:**
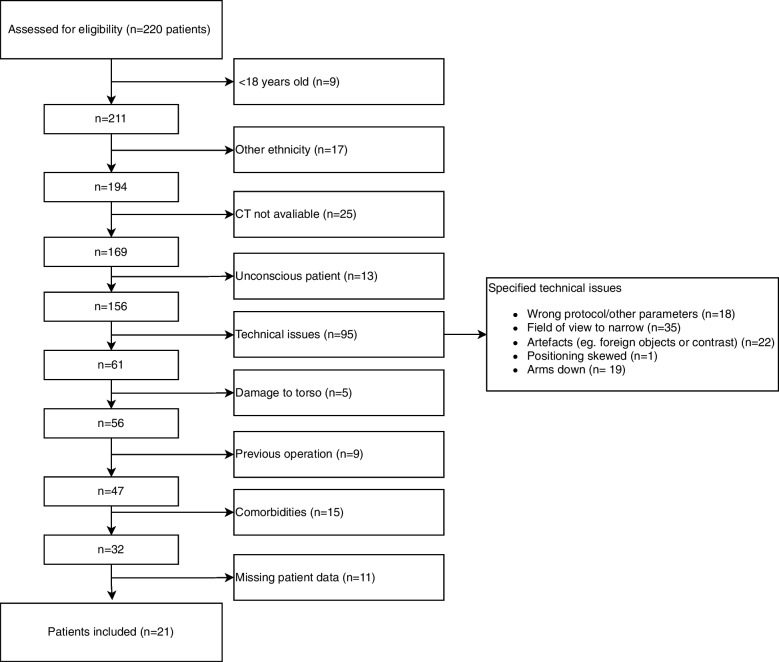
Flow diagram of the eligible, excluded, and included study patients

The trauma CT scans were selected to ensure a standardised scan protocol with intravenous contrast and a procedure with both thoracic and abdominal CT with presumable healthy patients. Figure 1 shows the inclusion of patients. Inclusion criteria were Caucasian patients ≥ 18 years old. The exclusion criteria were sign of circulatory shock, damage to the torso, or comorbidities (heart failure, chronic respiratory diseases, chronic kidney disease, chronic inflammatory diseases, endocrinological diseases, or cancer, as these diseases are known to affect the body composition) [5–7]. The comorbidities hypertension, hypercholesterolemia, and anxiety were allowed in the study. In addition, patients with previous thoracic and abdominal surgery, or foreign objects, such as metallic implants, were excluded due to the risk of scar tissue and artefacts.

### CT protocol

Trauma CT scans were identified by the Danish Medical Classification System. Scans were conducted using SOMATOM Definition Flash (Siemens Healthineers, Erlangen, Germany), Light Speed Pro32, or Discovery CT750HD (General Electric Healthcare, Chicago, IL, USA). All scanners were air calibrated daily and constancy tested monthly using producer fabricated water and air phantom.

Patients were in supine position with arms raised above the head and full inspiratory phase. The technical parameters were as follows: 120 kVp, autoregulated mAs, single collimation 0.6–0.625 mm, scan field of view 50 cm, total collimation width 20–40 mm, pitch 0.5–0.984, axial reconstruction, slice thickness 5 mm, and kernel Standard (General Electric scanners) or B31f (Siemens scanners).

Scans were conducted in venous contrast phase by the following steps: (i) bolus of 100 mL contrast (Iomeron 400 mg/mL, Bracco, Milan, Italy) with intravenous injection speed 3.0–4.1 mL/s, followed by a bolus of 37–50 mL saline with injection speed 2.9–4.1 mL/s, and (ii) bolus tracking with a region of interest set at the aorta at 1st lumbar vertebra level (the scan was initiated 30–35 s after HU increased by 100 HU). For further information on CT parameters, see Supplementary Table S2.

### Image selection and analysis

**Fig. 2:**
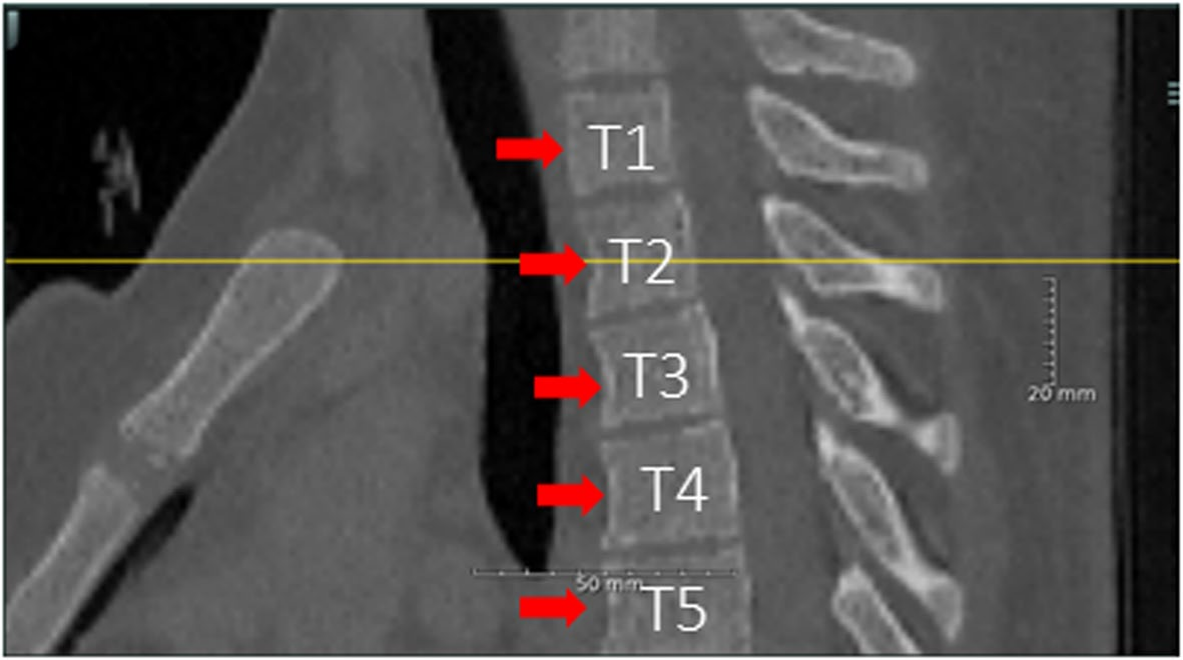
Selection of the axial image closest to the midline of the anterior margin of the vertebra (seen in sagittal view) was conducted. The red arrows show the anterior margins of the vertebras. The yellow line shows the midline of the vertebra where the axial image is selected. *T*, Thoracic vertebra

First, in eligible patients, both raters select axial images from the T2 to T12 and third lumbar level independently. Rater 1 selected all axial images from each level for all 21 patients, whereas rater 2 only selected all axial images for each level of ten patients chosen at random. The two raters selected images based on the following criteria: (i) the axial image closest to the midline of the anterior margin of the vertebra seen in sagittal view (Fig. 2) and (ii) in the case of a tie between two images, the one below the midline was chosen.

Second, images from the ten patients selected by both raters were checked for agreement of the image selections before any muscle assessment was conducted. If any disagreement on the selected axial images occurred, a consensus between the raters was made.

Third, both raters conducted muscle measures independently. Rater 1 conducted muscle measures on all images of the 21 patients, and rater 2 conducted muscle measures on the ten patients chosen at random from the image selection. The two raters both conducted a test–retest of the images of ten patients, independently. Rater 1 recorded the time spend on segmentation of all 21 patients the first time. Rater 1 had 9 months between measures, was a pulmonologist fellow with 4 years of experience, and had 2 years of experience and education within thoracic CT imaging. The secondary rater had 4 months between measurements, had 11 years of experience as a resident, and was a radiologist fellow with 10-month experience and education within thoracic CT imaging.

### Software and settings

**Fig. 3:**
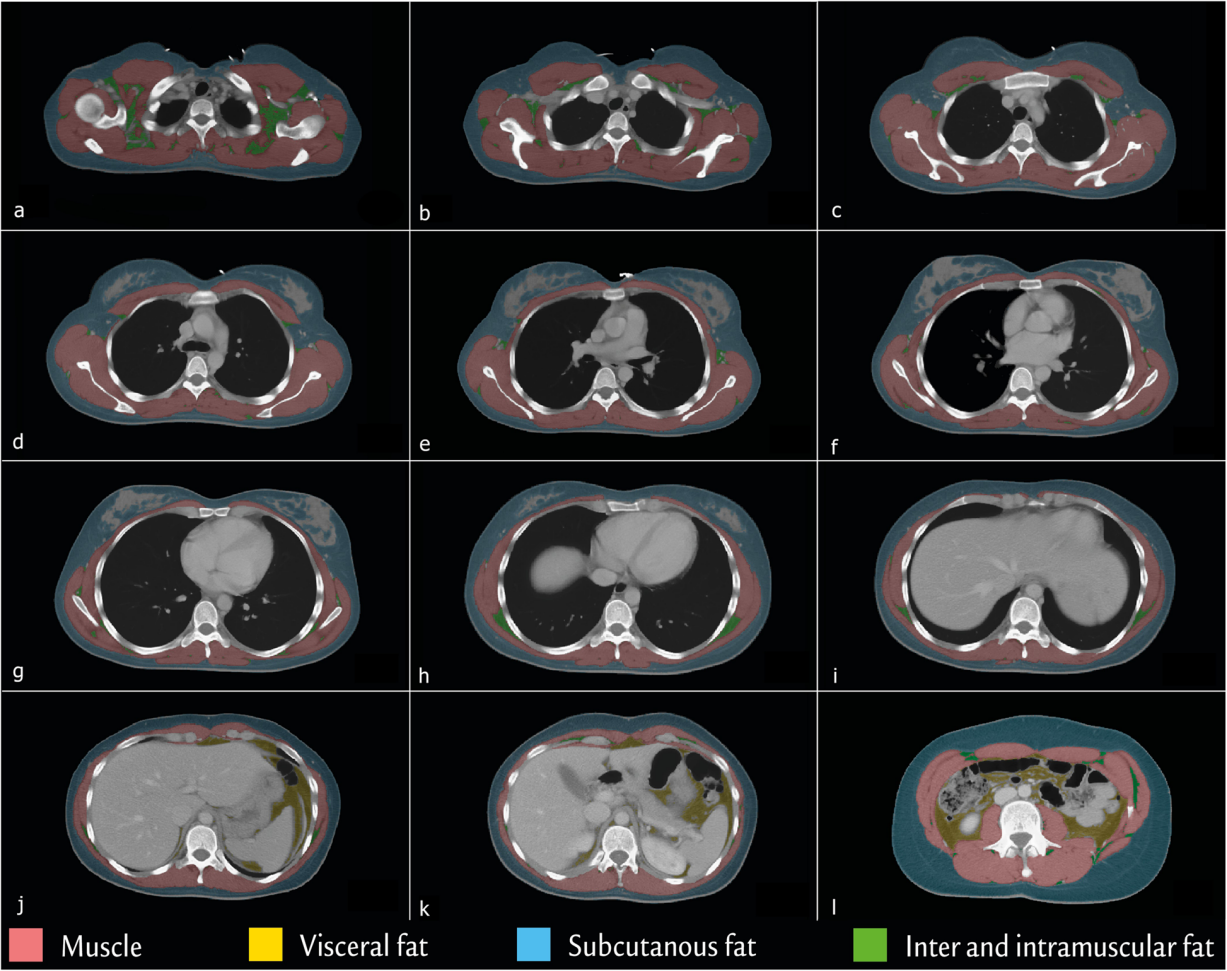
Example of segmentation of the axial images from a contrast-enhanced thoracic and abdominal computed tomography scan on a 27-year-old female. Thoracic level 2 (**a**), thoracic level 3 (**b**), thoracic level 4 (**c**), thoracic level 5 (**d**), thoracic level 6 (**e**), thoracic level 7 (**f**), thoracic level 8 (**g**), thoracic level 9 (**h**), thoracic level 10 (**i**), thoracic level 11 (**j**), thoracic level 12 (**k**), and lumbar level 3 (**l**)

A threshold-based semiautomated “Viking Slice” software was used for segmentation [24]. An example of segmentation can be seen in Fig. 3. The upper and lower threshold for muscle tissue attenuation were HU between -29 and + 150 HU, while they were -190 and -30 HU for fat tissue [24, 25].

When loading an image for analysis, the software makes a preliminary estimate based on the HU of the pixel as described above, hereby classifying pixels as either muscle, subcutaneous fat, visceral fat, and other tissues (bone and connective tissue). Minor corrections from the raters were necessary to remove blood vessels in the dermis and subcutaneous compartment, as well as larger vessels in axillary region of the thorax, and the intestine in the intraabdominal compartment as these were interpreted as muscle based on the HU. It was a simple manoeuvre allowing large areas to be corrected at a time and thereby removing pixels mistaken for muscle without interfering with pixels defined as fat or other tissues. Correction of each image takes approximately 2–7 min.

Data were reported as cumulated SMA (cm^2^) of a single axial image (not including fat) and the height-adjusted SMI (cm^2^/m^2^) from T2 to T12. T1 is left out due to these images consisted of neck and throat muscles in many patients. The intercostal muscles were excluded from the segmentation to avoid the effect of bone area and bone density from the costae on the SMA and SMD. The SMD was reported as the mean HU of measured SMA. The lower the mean HU, the poorer the muscle quality [25].

### Statistics

Data were reported descriptively. Demographics and skeletal muscle measurements are reported separately as median and 95% confidence interval (CI) for continuous variables and as proportions for categorical variables. Data was checked for normal distribution. The Wilcoxon-Mann–Whitney test was used to assess sex difference with ordinal data. A Pearson’s correlation was applied between the SMA, SMI, and SMD of each thoracic level and L3. The correlation of SMA, SMI, and SMD was considered stable as females are expected to have lower measurements than males.

Inter-rater agreement was evaluated by a two-way intraclass correlation coefficient (ICC) random-effect model, and intra-rater agreement was evaluated by the two-way ICC mixed-effect model. The individual ICC was reported in both the inter- and intra-rater agreement. Furthermore, Bland–Altman plots were used to visualise the agreement between raters and systematic bias along with test–retest.

Statistical analyses were conducted using STATA 17.0 (Stata Corp LLC, College Station, TX, USA).

## Results

In total, 21 patients (11 males, 10 females) were included in this proof-of-concept study. They had a median age of 29 years old (range 18 to 60 years). Males were significantly taller than females (median, males 1.78 m, females 1.68 m, *p* = 0.003). As shown in Table 1, males had significantly higher SMA (*p* < 0.0001–0.0009) and SMI (*p* = 0.0003–0.0015) than females at all thoracic levels and L3.

**Table 1:** The skeletal muscle area, skeletal muscle index, and skeletal muscle density

		Females			Males			*p*-value
	Level	Number	Median	95% *CI*	Number	Median	95% *CI*	
SMA (cm^2^)	T2	10	188.5	150.1–200.0	11	314.7	222.3–385.7	0.0001
	T3	10	176.6	128.8–209.2	11	298.3	195.6–350.3	0.0002
	T4	10	157.2	116.0–192.7	11	270.3	178.8–319.2	0.0002
	T5	10	141.2	102.9–168.6	11	242.6	152.4–296.2	0.0003
	T6	10	127.4	86.0–148.3	11	215.0	132.3–273.1	0.0003
	T7	10	107.2	69.4–127.9	11	177.2	107.1–228.1	0.0004
	T8	10	88.6	56.5–106.3	11	142.6	82.1–189.2	0.0006
	T9	10	70.2	46.5–90.0	11	126.7	82.3–165.7	0.0003
	T10	10	69.3	53.0–81.9	11	119.9	74.9–148.6	0.0002
	T11	9	64.9	52.7–80.3	11	115.5	72.2–155.4	0.0003
	T12	10	62.6	57.1–80.6	11	121.7	76.7–157.2	0.0001
	L3	10	117.1	111.3–144.2	11	202.1	139.0–225.3	0.0002
SMI (cm^2^/m^2^)	T2	10	70.4	57.2–73.5	11	97.8	70.2–119.0	0.0004
	T3	10	66.7	49.1–74.1	11	90.1	61.7–107.4	0.0007
	T4	10	59.2	44.2–68.3	11	79.1	56.4–97.3	0.0006
	T5	10	54.3	39.2–59.7	11	72.4	48.1–88.2	0.0009
	T6	10	48.6	32.8–52.5	11	64.9	41.8–78.9	0.0009
	T7	10	39.4	26.4–46.9	11	56.5	33.8–66.7	0.0015
	T8	10	32.7	21.5–39.0	11	45.1	25.9–54.7	0.0015
	T9	10	26.8	17.7–31.9	11	38.7	26.0–47.9	0.0012
	T10	10	24.8	19.8–29.0	11	37.1	24.0–45.3	0.0009
	T11	9	24.3	20.1–28.5	11	33.1	22.8–51.3	0.0012
	T12	10	23.8	19.8–28.6	11	37.6	24.2–45.3	0.0003
	L3	10	45.1	36.3–51.1	11	61.0	43.9–70.8	0.0006
SMD (mean HU)	T2	10	60.0	43.2–63.2	11	53.1	47.5–62.1	0.0486
	T3	10	60.1	46.4–64.5	11	52.4	47.3–61.5	0.0201
	T4	10	60.8	50.6–64.9	11	52.6	45.9–59.9	0.0015
	T5	10	60.2	49.3–65.5	11	51.4	40.6–56.6	0.0019
	T6	10	57.1	49.4–63.9	11	51.4	32.2–54.6	0.0008
	T7	10	54.8	48.9–62.2	11	47.0	30.9–54.9	0.0031
	T8	10	52.1	46.1–59.7	11	42.4	34.8–53.2	0.0060
	T9	10	53.7	47.0–59.5	11	43.9	32.2–56.4	0.0031
	T10	10	55.6	46.4–65.7	11	45.6	33.6–54.7	0.0006
	T11	9	56.3	45.4–61.9	11	45.5	34.3–57.1	0.0027
	T12	10	59.2	44.1–67.1	11	46.4	34.3–57.2	0.0011
	L3	10	59.6	42.5–72.7	11	50.0	40.1–60.1	0.0075

*p*-values are reported for the differences between males and females. *CI* Confidence interval, *HU* Hounsfield units, *SMA* Skeletal muscle area, *SMD* Skeletal muscle density, *SMI* Skeletal muscle index

### Body composition

**Fig. 4:**
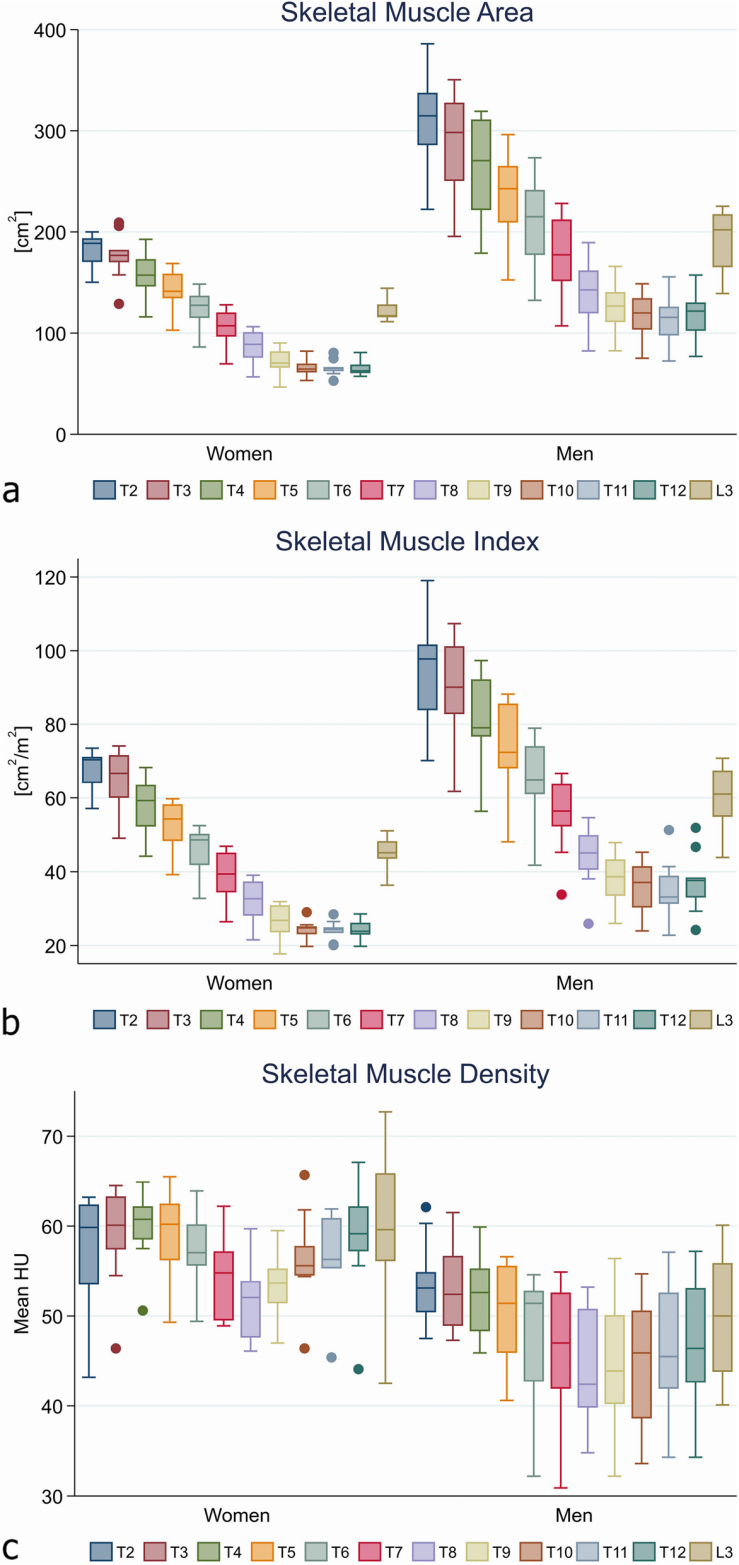
Distribution of muscle measures visualised with boxplots for each level for females and males. Skeletal muscle area (**a**). Skeletal muscle index (**b**). Skeletal muscle density (**c**)

Figure 4 demonstrates SMA (a), SMI (b), and SMD (c) at T 2–12 and L3, in males and females. Figure 4a shows that the thoracic level with largest SMA was T2 with a median of 314.7 cm^2^ for males and 188.5 cm^2^ for females. As seen in Fig. 4a, SMA decreased with each descending thoracic level for both males and females. Figure 4b shows that the same applied for the SMI, where males had highest median of 97.8 cm^2^/m^2^ and females had median of 70.4 cm^2^/m^2^ at T2, with decreasing SMI for each descending thoracic level. The SMI at L3 was 61.0 cm^2^/m^2^ for males and 45.1 cm^2^/m^2^ for females. Figure a and b shows that males had wider CI in both SMA and SMI, whereas females had a plateau at the lower thoracic levels T10–T12. SMA and SMI increased at L3 in both sexes and had a median of 202.1 cm^2^ and 117.1 cm^2^, respectively. As shown in Fig. 4c, females had higher median SMD compared to males. However, the widest CI was observed in females at L3 level. The level with the lowest median SMD was at T8 in males and females.

We found a linear association between SMA, SMI, and SMD at all thoracic levels and the L3. For further information, see Supplementary Figs. S3, S4 and S5.

### Correlations

As shown in Table 2, there was a significant correlation between SMA of each of the thoracic levels and the L3, ranging from *r* = 0.917 at T8 to *r* = 0.970 at T5 (all *p*-values < 0.0001). The same applied for the SMI, where there was a significant correlation between each of the thoracic levels and L3, ranging from *r* = 0.883 at T6 and T10 to *r* = 0.938 at T11 (all *p*-values < 0.0001). Furthermore, there was a significant correlation between the SMD of each thoracic level and L3 that varied from *r* = 0.526 at T2 to *r* = 0.890 at T10 (*p*-values between < 0.0001 and 0.0142).

**Table 2:** Correlation between the skeletal muscle area, skeletal muscle index, and skeletal muscle density at each of the thoracic levels T2 − T12 and the third lumbar level (L3)

	**SMA**		**SMI**		**SMD**	
Comparison	*r*	*p*	*r*	*p*	*r*	*p*
T2 *versus* L3	0.961	< 0.00001	0.912	< 0.00001	0.526	0.0142
T3* versus* L3	0.966	< 0.00001	0.903	< 0.00001	0.585	0.0054
T4 *versus* L3	0.968	< 0.00001	0.921	< 0.00001	0.751	0.0001
T5 *versus* L3	0.970	< 0.00001	0.924	< 0.00001	0.808	< 0.0001
T6 *versus* L3	0.944	< 0.00001	0.883	< 0.00001	0.776	< 0.0001
T7 *versus* L3	0.930	< 0.00001	0.869	< 0.00001	0.814	< 0.0001
T8 *versus* L3	0.917	< 0.00001	0.837	< 0.00001	0.878	< 0.0001
T9 *versus* L3	0.936	< 0.00001	0.849	< 0.00001	0.821	< 0.0001
T10 *versus* L3	0.960	< 0.00001	0.883	< 0.00001	0.890	< 0.0001
T11 *versus* L3	0.955	< 0.00001	0.938	< 0.00001	0.848	< 0.0001
T12 *versus* L3	0.953	< 0.00001	0.931	< 0.00001	0.886	< 0.0001

*SMA* Skeletal muscle area, *SMD* Skeletal muscle density, *SMI* Skeletal muscle index

### Agreement and reliability

The image selection showed there was high agreement between the two raters of 97%. There was consensus agreement on 3 single images out of the 120 images (2.5%) both raters selected. The inter-rater agreement of SMA was lowest at T11 with an ICC = 0.9940 and had the lowest lower limit (CI = 0.9770). All other levels had higher ICC and higher lower limit CI. The levels with the strongest interrater agreement were at T4, T5, and T7, all with ICC = 0.9994 (*p* < 0.0001).

Figure 4 a and b shows the inter- and intra-rater agreement of SMA of each thoracic level and the L3. The intra-rater agreement of SMA for the primary rater had the lowest agreement at T12, with ICC = 0.9908 (*p* < 0.0001), with the lowest lower limit CI = 0.9636 at T12. Also, the secondary rater had lowest agreement of SMA at T12 with ICC = 0.9961 (*p* < 0.0001), with the lowest lower limit of CI = 0.9844. The strongest intra-rater agreement for rater 1 was at T7 with ICC = 0.9998, whereas the secondary rater had the strongest intra-rater agreement at T2 and T4 with ICC = 0.9999 (see Supplementary Tables S6.1, S6.2 and S6.3 for further information on inter- and intra-rater ICC and CI).

**Fig. 5:**
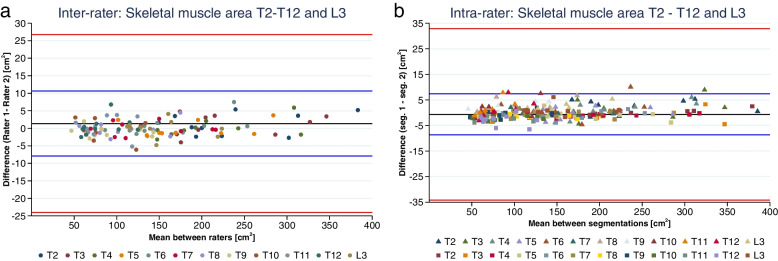
**a** The inter-rater agreement of the skeletal muscle area on each thoracic level and the third lumbar level. **b** The intra-rater agreement of the skeletal muscle area for rater 1 (triangle) and rater 2 (square). The blue lines show the 95% confidence interval; red lines show the 95% prediction interval

As seen in Fig. 5a, the differences in SMA between raters 1 and 2 are visualised based on the first segmentation. Figure 5b shows the differences between the first and the second segmentation in SMA. Most of the differences observed are gathered around 0 with random fluctuation. There were a few outliers. For further information on the Bland Altman plots, see Figs. S7 and S8.

## Discussion

To our knowledge, this is the first study that investigates skeletal muscle mass by systematic thoracic muscle segmentation from T2 to T12 in a study population without significant comorbidities, comparing each thoracic level to L3. Males had larger SMA and SMI compared to females, whereas females had the highest SMD at all thoracic and levels along with L3. There was an excellent correlation between the SMA of all thoracic levels and the L3; however, the strongest correlation was seen between T5 and L3. The correlation between the SMD at each thoracic level to the L3 was strong. The method proves valid when assessing skeletal muscle mass in presumed healthy Caucasian on contrast-enhanced thoracic CT as there was a high inter- and intra-rater agreement with the highest interrater agreements at T4, T5, and T7.

We found that the largest muscle mass in both sexes was found at T2, decreasing towards the lower thoracic levels, and SMA had, at all levels, an excellent correlation to L3. The strongest correlation to L3 was seen at the T5 level, which suggests this area may be most favourable to assess the SMA and SMI when only thoracic CT is available in terms of finding low or reduced SMA and SMI.

There are some discrepancies in current literature as to whether the SMA is affected by intravenous contrast. One study has shown that SMA may not be affected by the intravenous contrast though the SMD becomes higher [23]. Others suggest that both the SMA and SMD are slightly affected when using intravenous contrast [21, 22]. One could speculate that the muscle quality may be falsely enhanced when the radiodensity is used in contrast-enhanced CT. Previously, others have been looking at single muscle groups of the thoracic region validating the method and correlated to the whole-body skeletal muscle mass using algorithms [18, 26]. However, one could argue that a single muscle group may not be representative for the whole-body skeletal muscle mass or pathological changes. Furthermore, the skeletal muscle mass may rather be considered a continuum, and grading the severity of reduced muscle may be of higher importance than arbitrary cutoffs [25].

In our study, females had higher median SMD than males on all thoracic levels along with L3 level. A possible explanation could be due to the fixed intravenous contrast dose, and females are smaller than males, and females have a smaller blood volume to dilute the contrast medium and less tissue to be distributed to, and results in higher density [14]. In our study, the correlation between the SMD of each thoracic level to the L3 was strong; however, it varied from *r* = 0.526 at T2 to *r* = 0.890 at T10. However, as this is a proof-of-concept study, further studies with larger study populations are needed to elaborate factors on contrast distributions. Though, this study suggests that the T10 level may be most favourable when SMD is assessed, thus indicating that it may be necessary to assess both the T5 for SMA and the T10 for SMD.

Previously, the focus has been on abdominal CT in creating references for skeletal muscle measures based on healthy individuals [9, 10]. Studies that have used thoracic CT have sparse information on the CT parameters, making it difficult to reproduce and transfer to clinical practice [18]. Furthermore, in some studies, there is scarce information on the presence of comorbidities which is problematic as a long list of comorbidities is known to affect the body composition or no information on ethnicity [9, 18, 19, 26]. Ethnicity has proven important when assessing the body composition as significant ethnic differences have been addressed regarding the abdominal visceral fat. For example, Japanese populations have higher abdominal visceral fat relative to the abdominal subcutaneous fat compared to Caucasian which in part could explain the predisposition of metabolic diseases in Japanese populations [27]. This calls for ethnicity to be incorporated into the clinical tool as well. Other well-known clinical tools (*e.g.*, spirometry) have different normal ranges based on ethnicity [28].

The inter- and intra-rater agreement showed low bias as most of the differences showed a low mean difference with random fluctuations. This supports that there were small differences between raters and between first and second segmentation for each of the raters. This indicates that the muscle measuring technique is both valid and reliable in thoracic and abdominal areas based on the inter- and intra-rater agreement; however, there were a few outliers in the inter-rater agreement which indicated that there was a trend that rater 1 had higher muscle measures than the rater 2 when there was a high mean. The same applied for the intra-rater agreement, where there was a trend that rater 1 measured higher muscle measures in the first segmentation than the second segmentation compared to rater 2. This could be due to the different time span between the first and second measure, as rater 1 had 9 months between segmentations, whereas rater 2 had 4 months between segmentations. One could speculate that level of training may influence the outcome. As the method is novel, no systematic training exists, which also may influence the outcome. The inter- and intra-rater agreement results may be improved using systematic training sessions and exercises as proven favourable in both invasive procedures and surgical settings.

### Limitations and methodological considerations

There are limitations to this study. This is intended as a proof-of-concept study to whether skeletal muscle mass and density assessed with thoracic CT were associated with assessments at the abdominal level. The number of patients excluded based on CT technical issues was necessary to ensure compatibility between patients and eliminate potential systematic errors and confounders regarding the method. As a result, the study population is small, and the findings concerning the muscle measures may not be generalizable to a larger population until a larger data set is available. However, this study may help set the basis for future studies by narrowing down thoracic areas strongly associated with the abdominal muscle area.

Due to the small study population, this study did not adjust for age or BMI which may have an influence on the outcome [1, 8, 22, 27]. As such, future studies should include a distinction between different age groups, gender, BMI, and ethnicity [9, 10, 29, 30]. In addition, as this is a retrospective observational study, there is a risk that the study individuals may have undiagnosed comorbidities that could have affected the skeletal muscle mass. However, the risk is considered low as both medical records, prescriptions, and thoracic and abdominal CT have been assessed.

In the current version of the analysis software, images are manually downloaded from the picture archiving and communication system and transferred to the software before analysis, which is time demanding. It will require further software improvements before it is applicable for clinical use. The time requirements could be reduced further by applying fully automated software based on artificial intelligence, machine learning, and neural networks, as seen in other studies with abdominal segmentation [31, 32], thereby reducing the operator workload.

## Conclusions

This study showed that there is an association between the skeletal muscle mass and density assessed by contrast-enhanced thoracic CT and abdominal CT. Furthermore, this study suggested that the T5 level may be the most favourable to assess the SMA, the T11 to assess SMI, and the T10 level to assess the SMD of the thorax when using contrast-enhanced thoracic CT compared to the L3. However, further studies are needed to validate this on a larger scale with consideration of whole-body muscle mass, muscle function, age, gender, and ethnicity.

## Supplementary Information

**Additional file 1: Table S1.** Computed tomography frequency with and without intravenous contrast. **Table S2**. Computed tomography parameters. **Figure S3**. Figures of the association between skeletal muscle area of each thoracic level to the L3. **Figure S4.** Figures of the association between the skeletal muscle index of each thoracic level to the L3. **Fig. S5.** Figures of the association between the skeletal muscle density of each thoracic level to the L3. **Table S6.** Intraclass correlation of inter-rater agreement and intra-rater agreement. **Table S6.1.** Intraclass correlation of inter-rater agreement between the two rater’s measurements of skeletal muscle area. **Table S6.2.** Intraclass correlation of intra-rater agreement for rater 1’s measurements of skeletal muscle area. **Table S6.3.** Intraclass correlation of intra-rater agreement for rater 2’s measurements of skeletal muscle area. **Fig. S7.** Bland Altman plots of interrater agreement of skeletal muscle area measures. **Fig. S8.** Bland Altman plot of intra-rater agreement of skeletal muscle area measures.

## Data Availability

The dataset analysed during the current study is not publicly available but is available from the corresponding author on reasonable request.

## Abbreviations

BMI: Body mass index
CI: Confidence interval
COPD: Chronic obstructive pulmonary disease
CT: Computed tomography
ICC: Intraclass correlation coefficient
L: Lumbar vertebra level
SMA: Skeletal muscle area
SMD: Skeletal muscle density
SMI: Skeletal muscle index
T: Thoracic vertebra level
