# Supplementary material for: Association between thoracic and third lumbar CT-derived muscle mass and density in Caucasian patients without chronic disease: a proof-of-concept study
Source: Eur Radiol Exp. 2023 May 29;7:26. doi: 10.1186/s41747-023-00340-1 (PMC10225410; doi:10.1186/s41747-023-00340-1)
Supplement: Supplementary file 1 — Additional file 1: Table S1. Computed tomography frequency with and without intravenous contrast. Table S2. Computed tomography parameters. Figure S3. Figures of the association between skeletal muscle area of each thoracic level to the L3. Figure S4. Figures of the association between the skeletal muscle index of each thoracic level to the L3. Fig. S5. Figures of the association between the skeletal muscle density of each thoracic level to the L3. Table S6. Intraclass correlation of inter-rater agreement and intra-rater agreement. Table S6.1. Intraclass correlation of inter-rater agreement between the two rater’s measurements of skeletal muscle area. Table S6.2. Intraclass correlation of intra-rater agreement for rater 1’s measurements of skeletal muscle area. Table S6.3. Intraclass correlation of intra-rater agreement for rater 2’s measurements of skeletal muscle area. Fig. S7. Bland Altman plots of interrater agreement of skeletal muscle area measures. Fig. S8. Bland Altman plot of intra-rater agreement of skeletal muscle area measures. [file 41747_2023_340_MOESM1_ESM.pdf]

ELECTRONIC SUPPLEMENTARY MATERIAL

Association between thoracic and third lumbar CT-derived muscle mass and density in Caucasian patients without chronic disease: a proof-of-concept study

Table of content

STROBE checklist....................2
S1. Computed tomography frequency with and without intravenous contrast....................4
S2. Computed tomography parameters....................5
S3. Figures of the association between skeletal muscle area of each thoracic level to the L3....................6
S4. Figures of the association between the skeletal muscle index of each thoracic level to the L3....................8
S5. Figures of the association between the skeletal muscle density of each thoracic level to the L3....................10
S6. Intraclass correlation of inter-rater agreement and intra-rater agreement....................12
S7. Bland Altman plots of interrater agreement of skeletal muscle area measures....................14
S8. Bland Altman plot of intra-rater agreement of skeletal muscle area measures....................16

ELECTRONIC SUPPLEMENTARY MATERIAL

STROBE checklist

	Item No	Recommendation	Page No
**Title and abstract**	1	(*a*) Indicate the study's design with a commonly used term in the title or the abstract<br>(*b*) Provide in the abstract an informative and balanced summary of what was done and what was found	1
**Introduction**			
Background/rationale	2	Explain the scientific background and rationale for the investigation being reported	3+4
Objectives	3	State specific objectives, including any prespecified hypotheses	4
**Methods**			
Study design	4	Present key elements of study design early in the paper	5
Setting	5	Describe the setting, locations, and relevant dates, including periods of recruitment, exposure, follow-up, and data collection	5+6
Participants	6	(*a*) Give the eligibility criteria, and the sources and methods of selection of participants. Describe methods of follow-up<br>(*b*) For matched studies, give matching criteria and number of exposed and unexposed	5+6
Variables	7	Clearly define all outcomes, exposures, predictors, potential confounders, and effect modifiers. Give diagnostic criteria, if applicable	5-+7
Data sources/ measurement	8*	For each variable of interest, give sources of data and details of methods of assessment (measurement). Describe comparability of assessment methods if there is more than one group	6
Bias	9	Describe any efforts to address potential sources of bias	
Study size	10	Explain how the study size was arrived at	5
Quantitative variables	11	Explain how quantitative variables were handled in the analyses. If applicable, describe which groupings were chosen and why	7
Statistical methods	12	(*a*) Describe all statistical methods, including those used to control for confounding<br>(*b*) Describe any methods used to examine subgroups and interactions<br>(*c*) Explain how missing data were addressed<br>(*d*) If applicable, explain how loss to follow-up was addressed<br>(*e*) Describe any sensitivity analyses	7
**Results**			
Participants	13*	(a) Report numbers of individuals at each stage of study—eg numbers potentially eligible, examined for eligibility, confirmed eligible, included in the study, completing follow-up, and analysed<br>(b) Give reasons for non-participation at each stage<br>(c) Consider use of a flow diagram	7+ figure 1
Descriptive data	14*	(a) Give characteristics of study participants (eg demographic, clinical, social) and information on exposures and potential confounders<br>(b) Indicate number of participants with missing data for each variable of interest<br>(c) Summarise follow-up time (eg, average and total amount)	7
Outcome data	15*	Report numbers of outcome events or summary measures over time	

Main results	16	(*a*) Give unadjusted estimates and, if applicable, confounder-adjusted estimates and their precision (eg, 95% confidence interval). Make clear which confounders were adjusted for and why they were included<br><br>(*b*) Report category boundaries when continuous variables were categorized<br>(*c*) If relevant, consider translating estimates of relative risk into absolute risk for a meaningful time period	7-9+ table 1+ Fig.4
Other analyses	17	Report other analyses done—eg analyses of subgroups and interactions, and sensitivity analyses	8+9+ table2 +fig.5+ S2-S7
**Discussion**			
Key results	18	Summarise key results with reference to study objectives	10
Limitations	19	Discuss limitations of the study, taking into account sources of potential bias or imprecision. Discuss both direction and magnitude of any potential bias	12
Interpretation	20	Give a cautious overall interpretation of results considering objectives, limitations, multiplicity of analyses, results from similar studies, and other relevant evidence	10-12
Generalisability	21	Discuss the generalisability (external validity) of the study results	12
**Other information**			
Funding	22	Give the source of funding and the role of the funders for the present study and, if applicable, for the original study on which the present article is based	24

*Give information separately for exposed and unexposed groups.

Note: An Explanation and Elaboration article discusses each checklist item and gives methodological background and published examples of transparent reporting. The STROBE checklist is best used in conjunction with this article (freely available on the Web sites of PLoS Medicine at http://www.plosmedicine.org/, Annals of Internal Medicine at http://www.annals.org/, and Epidemiology at http://www.epidem.com/). Information on the STROBE Initiative is available at http://www.strobe-statement.org.

ELECTRONIC SUPPLEMENTARY MATERIAL

S1. Computed tomography frequency with and without intravenous contrast.

Table S1 shows the number of solely thoracic CT 01.01.2021 to 30.06.2022 in the local University Hospital.

Type of thoracic computed tomography	Number of procedures (%)
Thoracic CT<br>+ Intravenous contrast<br>SKS code: UXCC00 + UXZ10	1573 (53.3%)
Thoracic CT + No intravenous contrast<br>SKS code: UXCC00 + UXZ11	1376 (46.7%)

SKS; The Danish Medical Classification system.

ELECTRONIC SUPPLEMENTARY MATERIAL

S2. Computed tomography parameters

Table S2 shows the scanner types and the scan parameters used for each of the scanner

	Siemens Somatom Definition Flash	GE light speed pro32	GE Discovery CT750HD
No. study individuals	2	5	14
CT parameters			
Kilovolt (kV)	120	120	120
mAs	Autoregulated	Autoregulated	Autoregulated
Pitch	0.5	0.969	0.984
Single collimation (mm)	0.6	0.625	0.625
Total collimation (mm)	38.4	40	20
Reconstruction slice thickness (mm)	5	5	5
Kernel	B31f	Standard	Standard
Scan field of view (cm)	50.0	50.0	50.0
Contrast media and bolus tracking			
1. Intravenous contrast media	100 mL	100 mL	100 mL
Iomeron (400mg/ml)	3.9-4.0	4.0-4.1	3.0-4.1
Injection speed (mL/sec)	40-50	40	37-50
2. Bolus saline (mL)	2.9-4.1	2.9-4.1	2.9-4.1
Saline injection speed (mL/sec)			
3. Bolus tracking was used ROI was set at the Aorta	1st lumbar vertebra level	1st lumbar vertebra level	1st lumbar vertebra level
4. Fixed delay; after bolus tracking (s)	30-35 sec	30-35 sec	30-35 sec

ROI: Region of interest

ELECTRONIC SUPPLEMENTARY MATERIAL

S3. Figures of the association between skeletal muscle area of each thoracic level to the L3

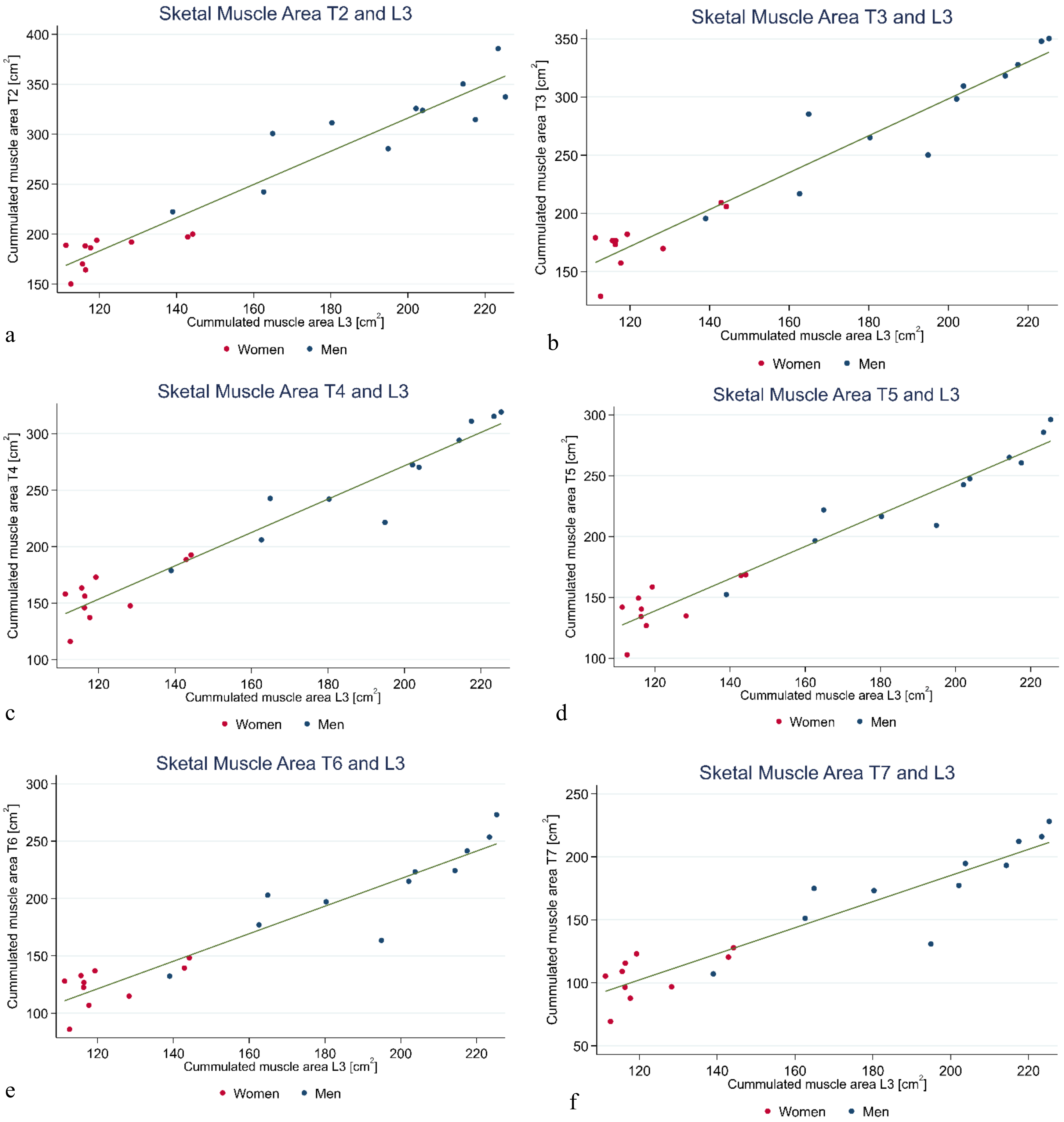

Continues next page

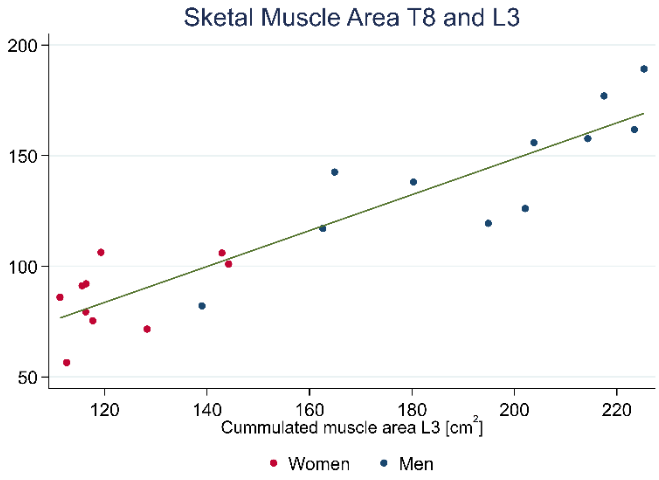

g

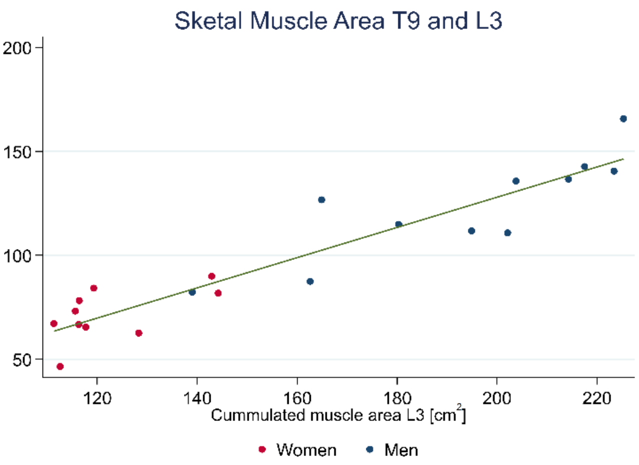

h

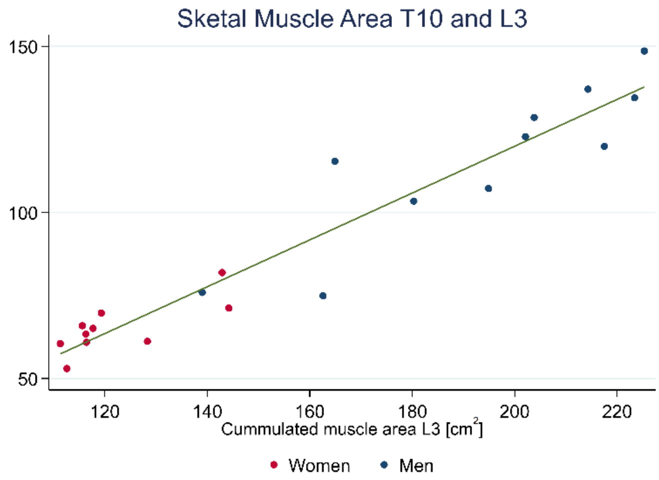

i

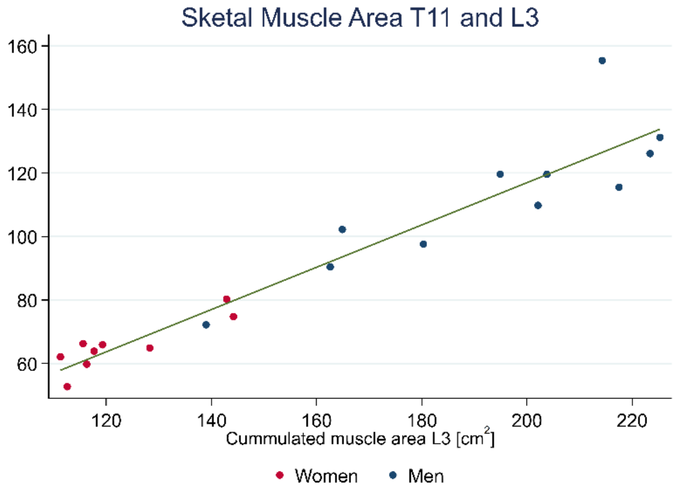

j

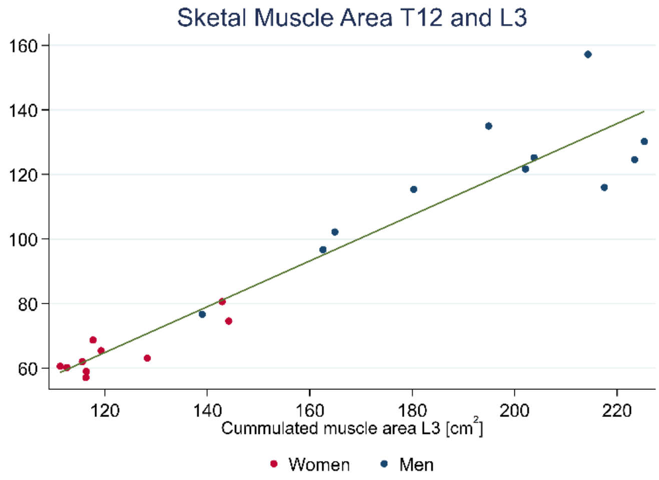

k

Figure S3 shows the scatters plots of skeletal muscle area for each thoracic level and the third lumbar level: thoracic level 2 and L3 (**a**), thoracic level 3 and L3 (**b**), thoracic level 4 and L3 (**c**), thoracic level 5 and L3 (**d**), thoracic level 6 and L3 (**e**), thoracic level 7 and L3 (**f**), thoracic level 8 and L3 (**g**), thoracic level 9 and L3 (**h**), thoracic level 10 and L3 (**i**), thoracic level 11 and L3 (**j**), thoracic level 12 and L3 (**k**). *L* lumbar level *T* thoracic level

ELECTRONIC SUPPLEMENTARY MATERIAL

S4. Figures of the association between the skeletal muscle index of each thoracic level to the L3

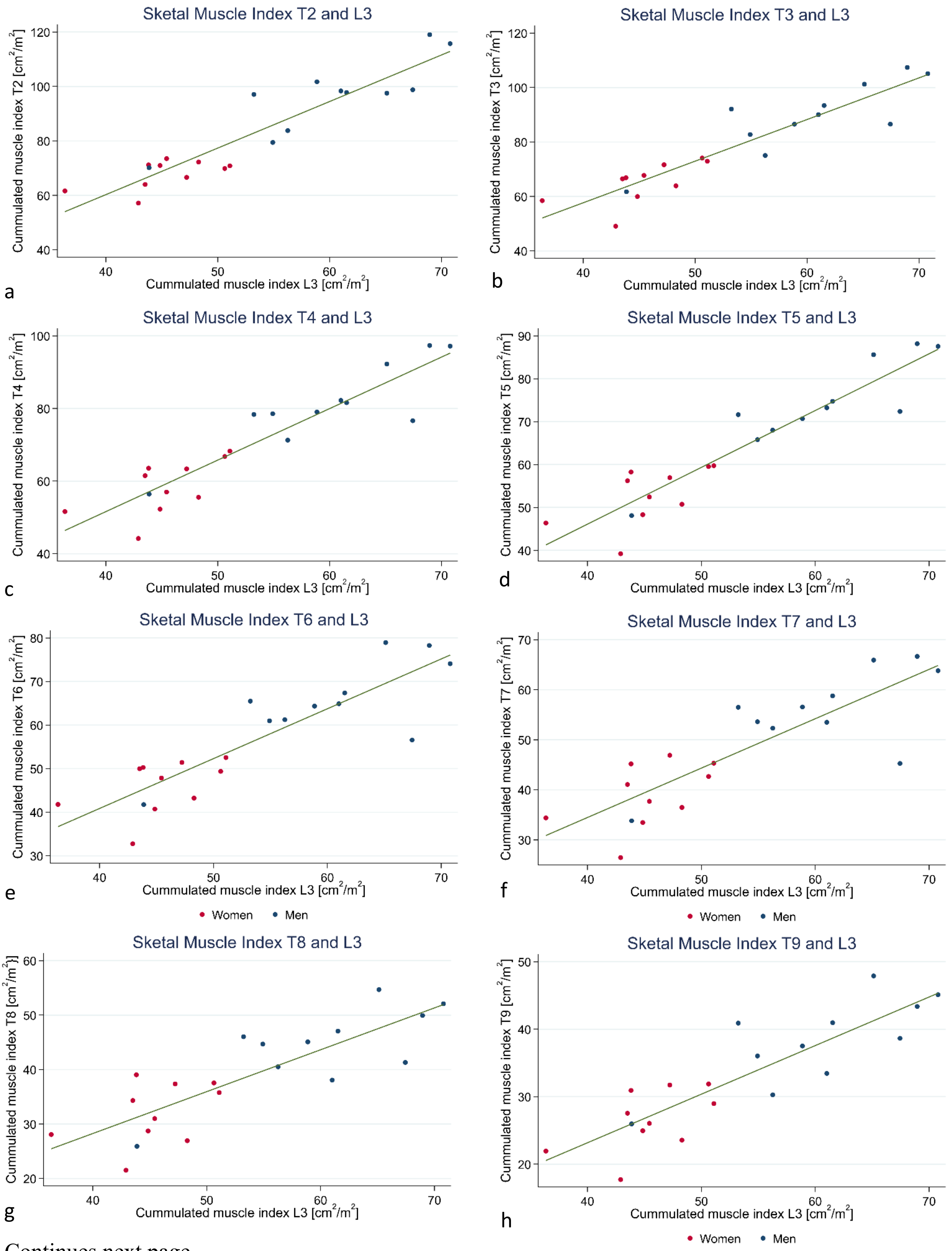

Continues next page

ELECTRONIC SUPPLEMENTARY MATERIAL

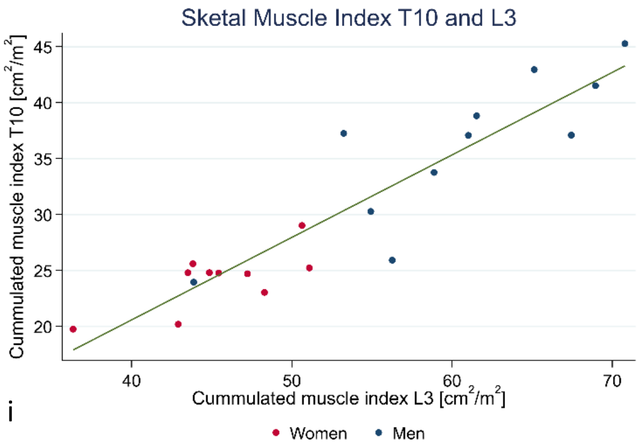

i

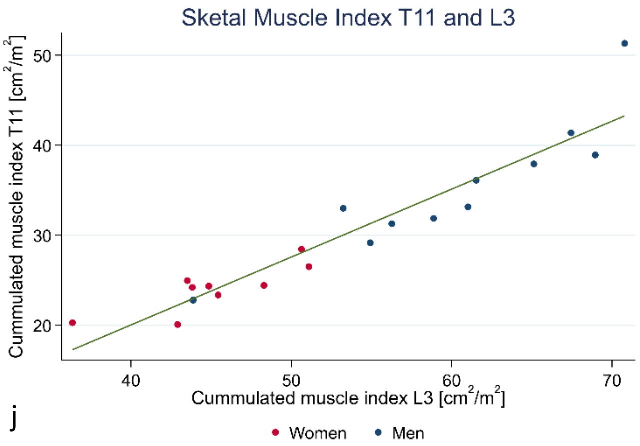

j

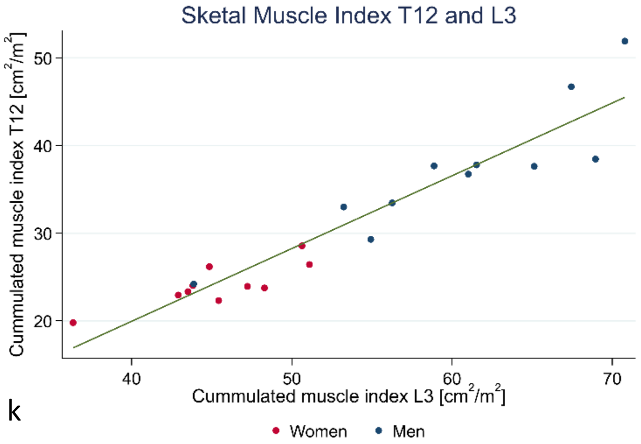

k

Figure S4 shows the scatters plots of skeletal muscle index for each thoracic level and the third lumbar level: thoracic level 2 and L3 (**a**), thoracic level 3 and L3 (**b**), thoracic level 4 and L3 (**c**), thoracic level 5 and L3 (**d)**, thoracic level 6 and L3 (**e**), thoracic level 7 and L3 (**f**), thoracic level 8 and L3 (**g**), thoracic level 9 and L3 (**h**), thoracic level 10 and L3 (**i**), thoracic level 11 and L3 (**j**), thoracic level 12 and L3 (**k**). *L* lumbar level *T* thoracic level

ELECTRONIC SUPPLEMENTARY MATERIAL

S5. Figures of the association between the skeletal muscle density of each thoracic level to the L3.

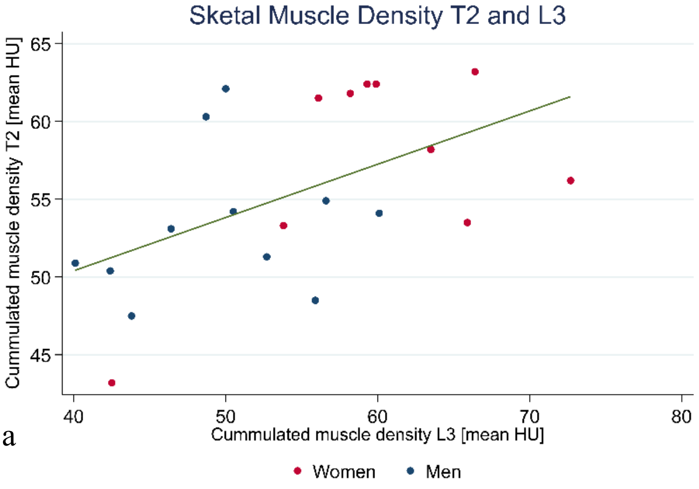

a

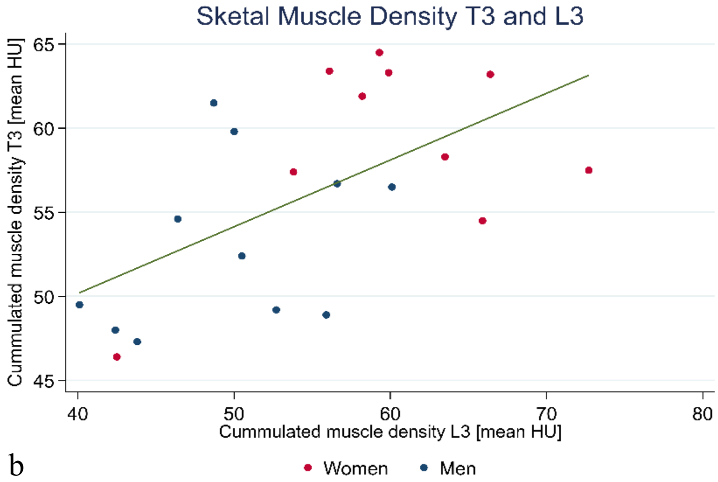

b

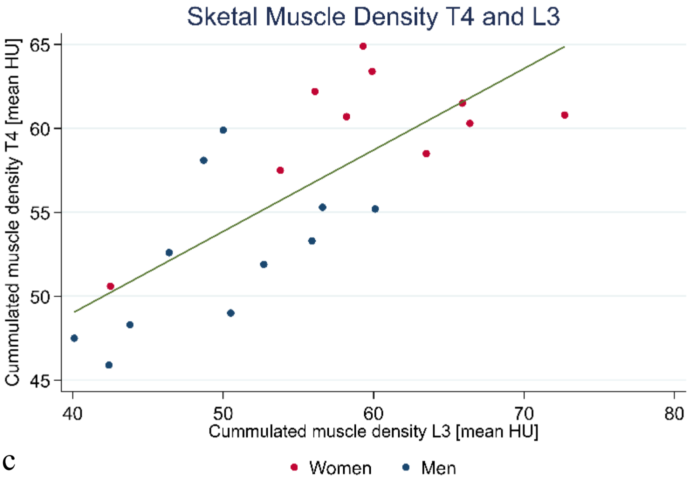

c

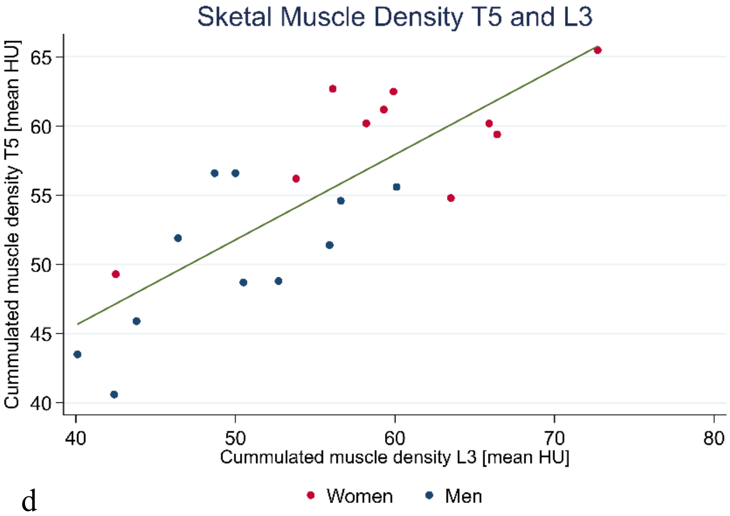

d

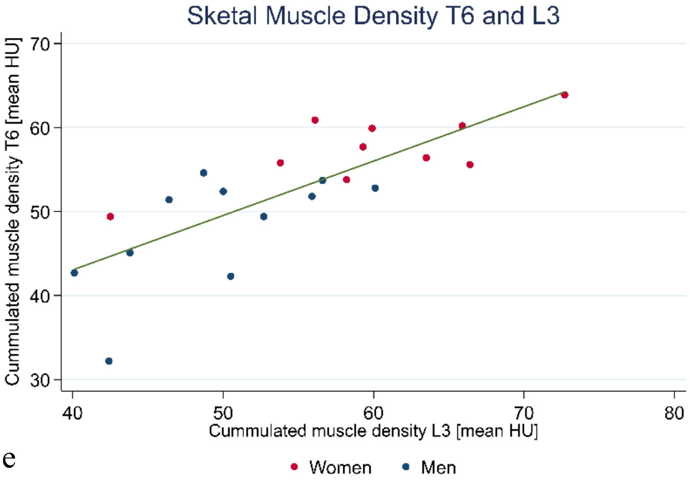

e

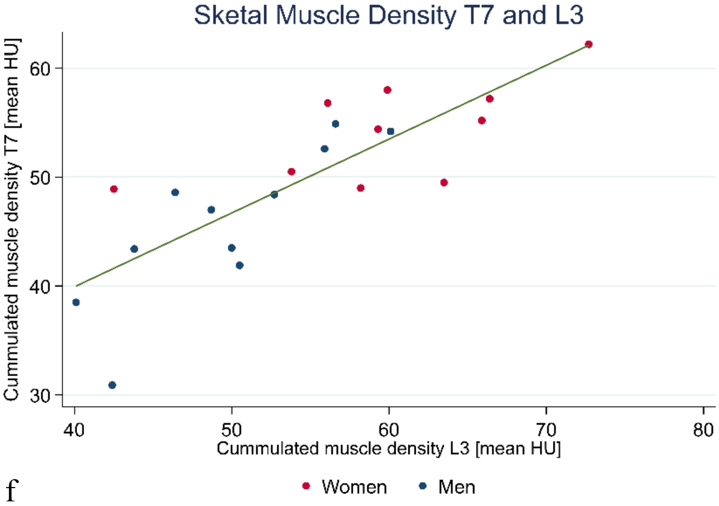

f

Continues next page

ELECTRONIC SUPPLEMENTARY MATERIAL

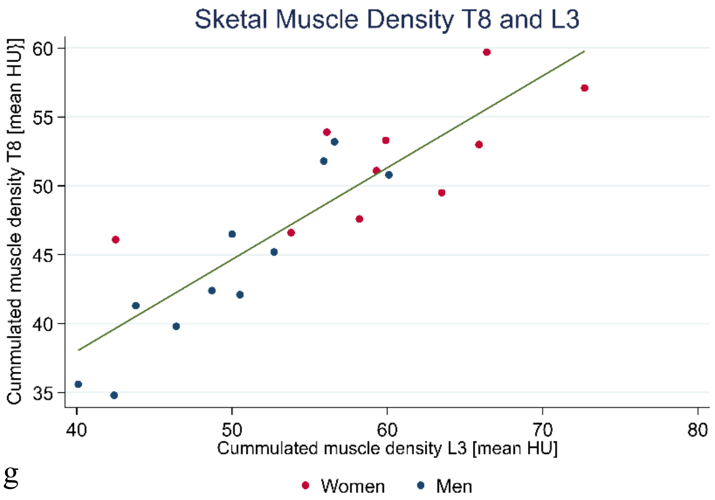

g

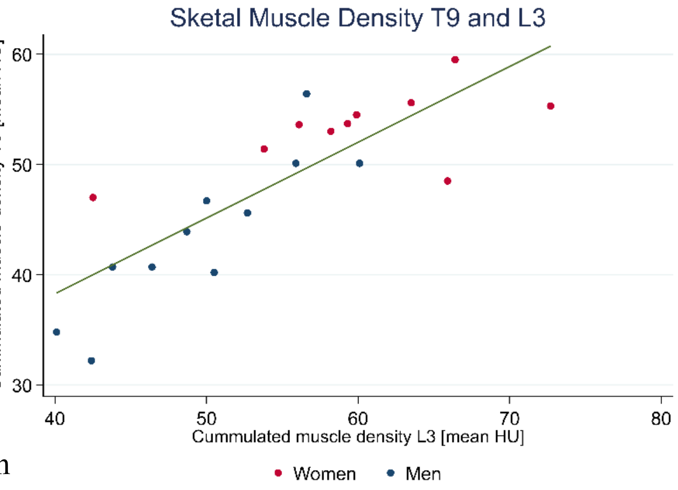

h

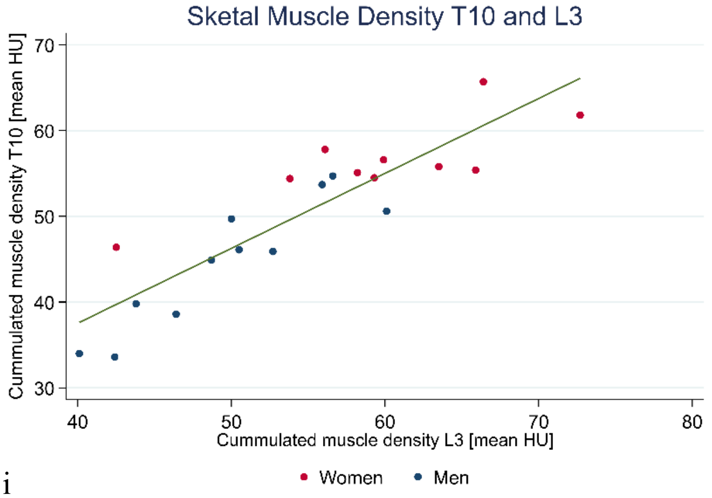

i

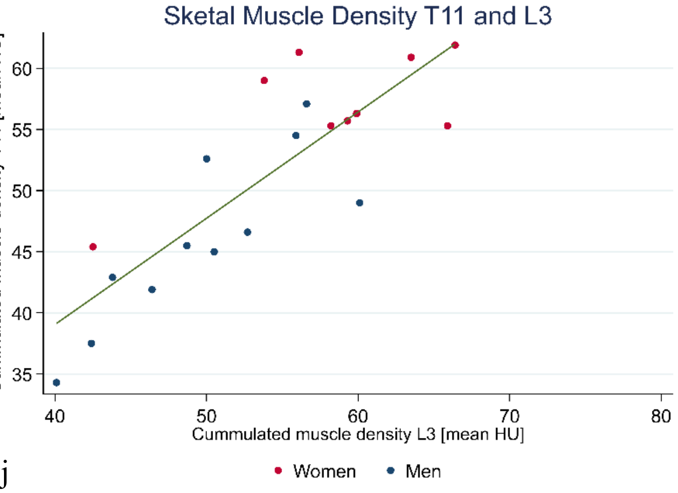

j

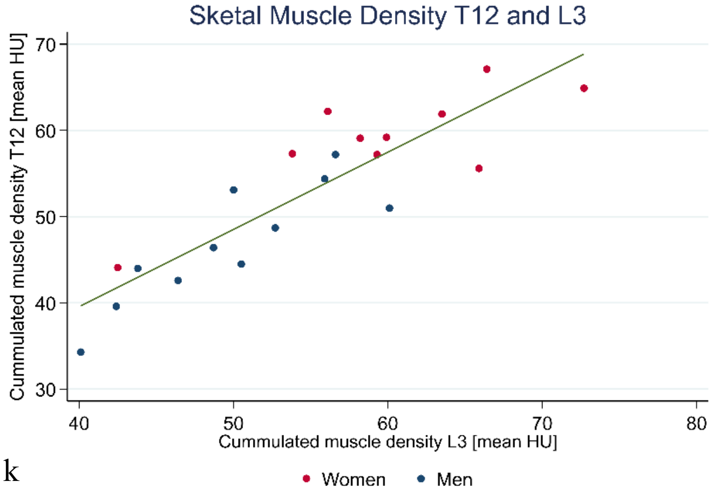

k

Figure S5 shows the scatters plots of skeletal muscle density for each thoracic level and the third lumbar level: thoracic level 2 and L3 (**a**), thoracic level 3 and L3 (**b**), thoracic level 4 and L3 (**c**), thoracic level 5 and L3 (**d**), thoracic level 6 and L3 (**e**), thoracic level 7 and L3 (**f**), thoracic level 8 and L3 (**g**), thoracic level 9 and L3 (**h**), thoracic level 10 and L3 (**i**), thoracic level 11 and L3 (**j**), thoracic level 12 and L3 (**k**). *L* lumbar level *T* thoracic level

S6. Intraclass correlation of inter-rater agreement and intra-rater agreement

Table S6.1 shows the intraclass correlation of inter-rater agreement between the two rater's measurements of skeletal muscle area for ten study individuals - first segmentation.

Inter -rater<br>Thoracic SMA	N	ICC	*95%CI*	*P*
T2	10	0.9991	(0.9965 - .9998)	<0.0001
T3	10	0.9992	(0.9970 - .9998)	<0.0001
T4	10	0.9994	(0.9975 - .9998)	<0.0001
T5	10	0.9994	(0.9975 - .9998)	<0.0001
T6	10	0.9989	(0.9959 - .9997)	<0.0001
T7	10	0.9994	(0.9977 - .9999)	<0.0001
T8	10	0.9984	(0.9938 - .9996)	<0.0001
T9	10	0.9988	(0.9933 - .9997)	<0.0001
T10	10	0.9954	(0.9825 - .9988)	<0.0001
T11	10	0.9940	(0.9770 - .9985)	<0.0001
T12	10	0.9949	(0.9802 - .9987)	<0.0001
L3	10	0.9985	(0.9939 - .9996)	<0.0001

CI: Confidence interval*; ICC:* Intra class correlation coefficient; *N:* Number of study individuals;

P: P-value; SMA: Skeletal muscle area

Table S6.2 shows the intraclass correlation of intra-rater agreement for rater 1's measurement of skeletal muscle area for ten study individuals.

Test-retest- rater 1<br>Thoracic SMA	N	ICC	*95%CI*	*P*
T2	10	0.9992	(0.9969 - 0.9998)	<0.0001
T3	10	0.9986	(0.9945 - 0.9997)	<0.0001
T4	10	0.9989	(0.9956 - 0.9997)	<0.0001
T5	10	0.9993	(0.9971 – 0.9999)	<0.0001
T6	10	0.9977	(0.9908 – 0.9994)	<0.0001
T7	10	0.9998	(0.9990 - 0.9999)	<0.0001
T8	10	0.9967	(0.9866 – 0.9992)	<0.0001
T9	10	0.9980	(0.9920 – 0.9995)	<0.0001
T10	10	0.9950	(0.9799 - 0.9987)	<0.0001
T11	10	0.9925	(0.9925 - 0.9981)	<0.0001
T12	10	0.9908	(0.9636 - 0.9977)	<0.0001
L3	10	0.9991	(0.9963 - 0.9998)	<0.0001

CI: Confidence interval*; ICC:* Intra class correlation coefficient; *N:* Number of study individuals;

P: P-value; SMA: Skeletal muscle area

Table S6.3 shows the intraclass correlation of intra-rater agreement for rater 2's measurements of skeletal muscle area (SMA) for ten study individuals.

Test-retest- rater 2<br>Thoracic SMA	N	ICC	*95%CI*	*P*
T2	10	0.9999	(0.9996 – 1.000)	<0.0001
T3	10	0.9996	(0.9984 - 0.9999)	<0.0001
T4	10	0.9999	(0.9998 – 1.0000)	<0.0001
T5	10	0.9996	(0.9983 – 0.9999)	<0.0001
T6	10	0.9996	(0.9984 - 0.9999)	<0.0001
T7	10	0.9996	(0.9985 - 0.9999)	<0.0001
T8	10	0.9998	(0.9990 – 0.9999)	<0.0001
T9	10	0.9990	(0.9961 - 0.9998)	<0.0001
T10	10	0.9992	(0.9968 - 0.9998)	<0.0001
T11	10	0.9994	(0.9975 - 0.9998)	<0.0001
T12	10	0.9961	(0.9844 - 0.9990)	<0.0001
L3	10	0.9989	(0.9954 - 0.9997)	<0.0001

CI: Confidence interval; *ICC:* Intra class correlation coefficient; *N:* Number of study individuals; *P: P-value; SMA:* Skeletal muscle area

S7. Bland Altman plots of interrater agreement of skeletal muscle area measures

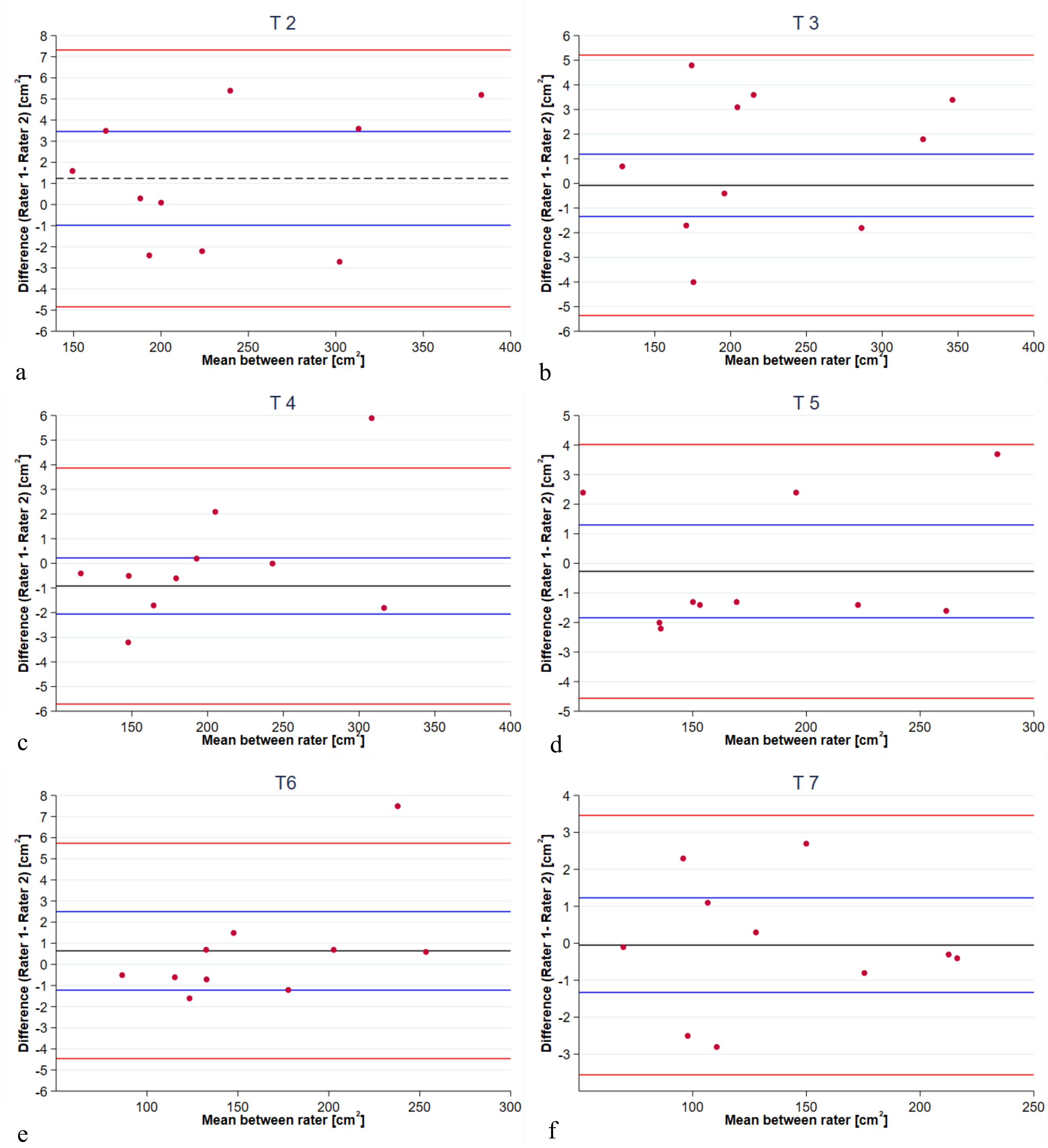

Continues next page

ELECTRONIC SUPPLEMENTARY MATERIAL

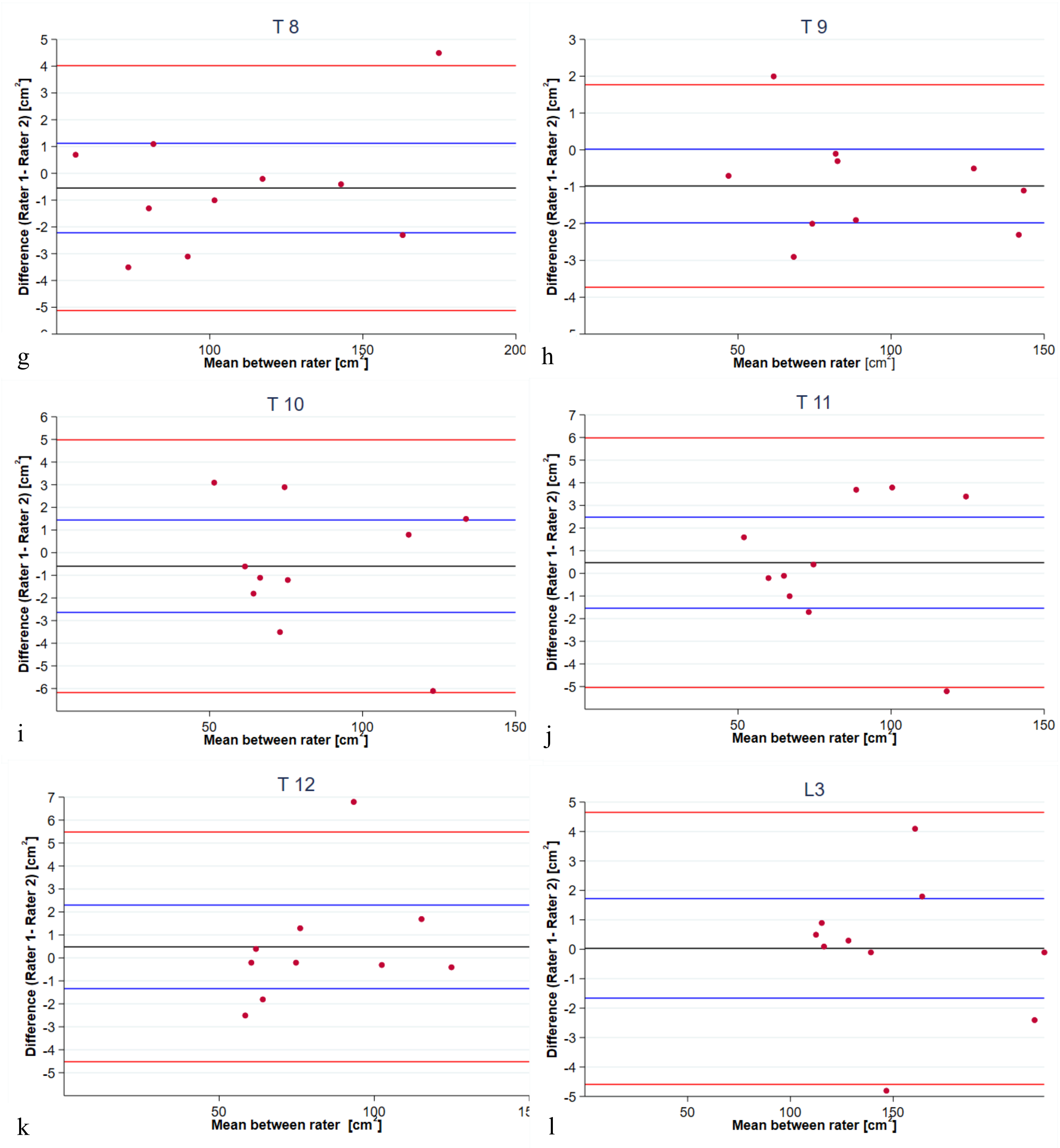

Figure S7 shows the interrater agreement between rater 1 and rater 2 of SMA for each thoracic level and third lumbar level using the Bland Altmann plots: thoracic level 2 (**a**), thoracic level 3 (**b**), thoracic level 4 (**c**), thoracic level 5 (**d**), thoracic level 6 (**e**), thoracic level 7 (**f**), thoracic level 8 (**g**), thoracic level 9 (**h**), thoracic level 10 (**i**), thoracic level 11 (**j**), thoracic level 12 (**k**), and lumbar level 3 (**l**). Measures from the 1st segmentation was used. The black line is the mean difference, blue lines are the 95% confidence interval, and red lines are the 95% prediction interval.

S8. Bland Altman plot of intra-rater agreement of skeletal muscle area measures

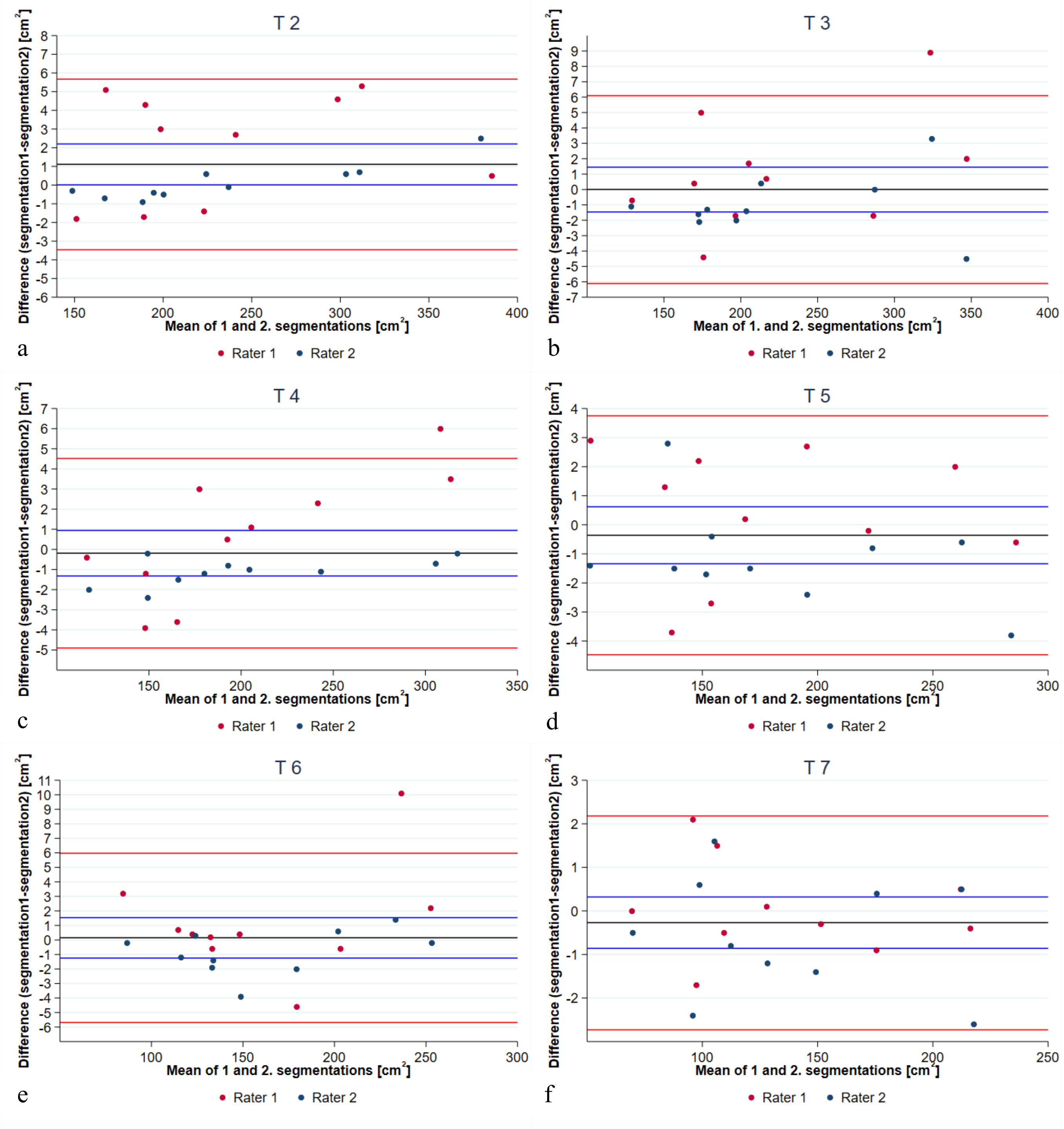

Continues next page

ELECTRONIC SUPPLEMENTARY MATERIAL

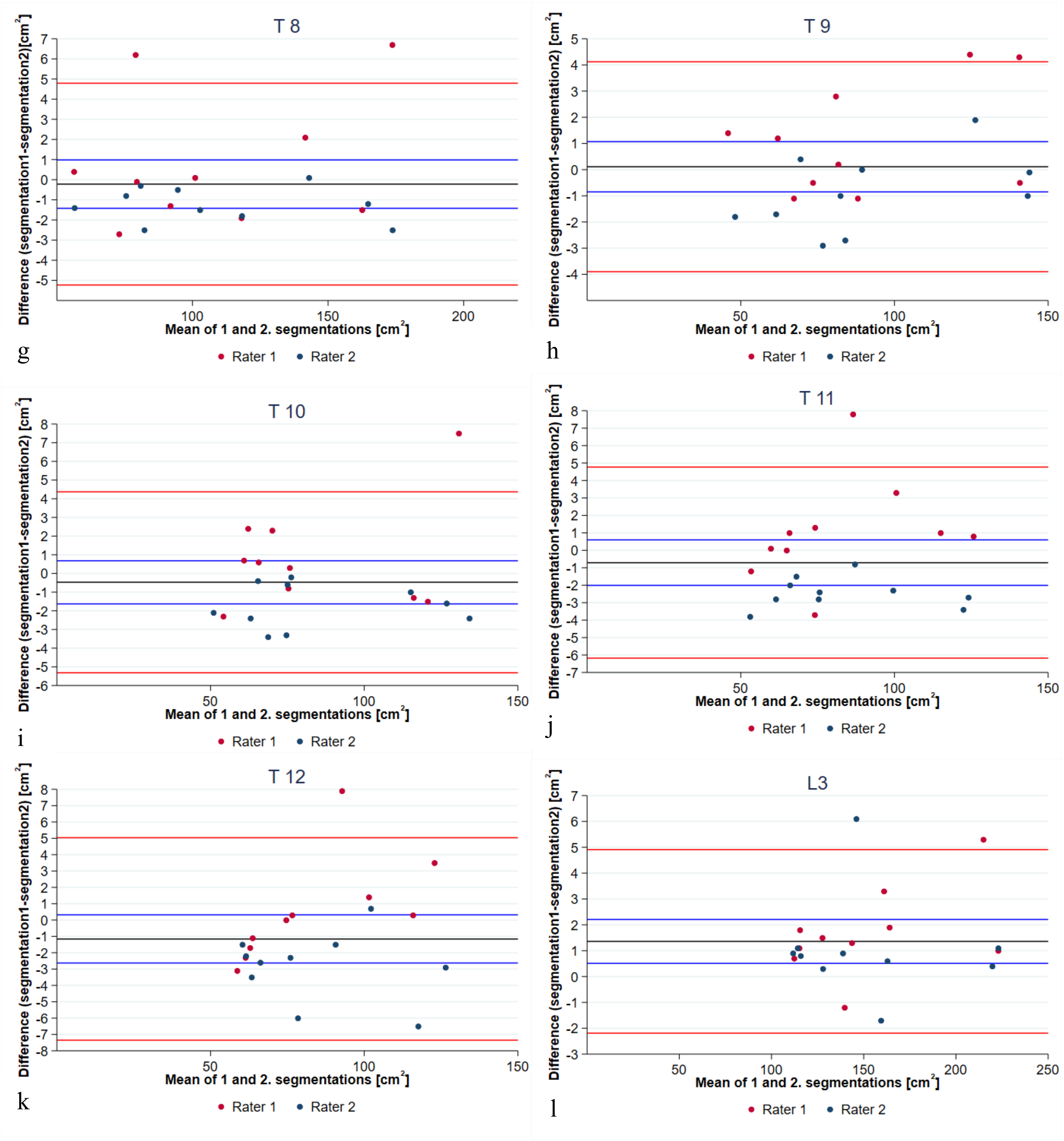

Figure S8 show the skeletal muscle area between the 1st and 2nd segmentation for rater 1 (red dots) and rater 2 (blue dots) for each thoracic level and the third lumbar level: thoracic level 2 (**a**), thoracic level 3 (**b**), thoracic level 4 (**c**), thoracic level 5 (**d**), thoracic level 6 (**e**), thoracic level 7 (**f**), thoracic level 8 (**g**), thoracic level 9 (**h**), thoracic level 10 (**i**), thoracic level 11 (**j**), thoracic level 12 (**k**), and lumbar level 3 (**l**). The black line is the mean difference, blue lines are the 95% confidence interval, and red lines are the 95% prediction interval.
